# Integrated climate-chemical indicators of diffuse pollution from land to water

**DOI:** 10.1038/s41598-018-19143-1

**Published:** 2018-01-17

**Authors:** Per-Erik Mellander, Phil Jordan, Marianne Bechmann, Ophélie Fovet, Mairead M. Shore, Noeleen T. McDonald, Chantal Gascuel-Odoux

**Affiliations:** 1 Department of Environment, Soils and Landuse, Teagasc, Johnstown Castle Environment Research Centre, Wexford, Ireland; 2 School of Geography and Environmental Sciences, Ulster University, Coleraine, Northern Ireland; 3 NIBIO, Environment and Natural Resources, Aas, Norway; 4 UMR SAS, INRA, Agrocampus Ouest, Rennes, France; 5 Environment Section, Wexford County Council, Carricklawn, Wexford, Ireland; 6 Agricultural Catchments Programme, Teagasc, Johnstown Castle Environment Research Centre, Wexford, Ireland

## Abstract

Management of agricultural diffuse pollution to water remains a challenge and is influenced by the complex interactions of rainfall-runoff pathways, soil and nutrient management, agricultural landscape heterogeneity and biogeochemical cycling in receiving water bodies. Amplified cycles of weather can also influence nutrient loss to water although they are less considered in policy reviews. Here, we present the development of climate-chemical indicators of diffuse pollution in highly monitored catchments in Western Europe. Specifically, we investigated the influences and relationships between weather processes amplified by the North Atlantic Oscillation during a sharp upward trend (2010–2016) and the patterns of diffuse nitrate and phosphorus pollution in rivers. On an annual scale, we found correlations between local catchment-scale nutrient concentrations in rivers and the influence of larger, oceanic-scale climate patterns defined by the intensity of the North Atlantic Oscillation. These influences were catchment-specific showing positive, negative or no correlation according to a typology. Upward trends in these decadal oscillations may override positive benefits of local management in some years or indicate greater benefits in other years. Developing integrated climate-chemical indicators into catchment monitoring indicators will provide a new and important contribution to water quality management objectives.

## Introduction

The integration of systems science is recognized as a global challenge for sustainability^1^. However, research that links water quality with climate is yet scarce due to their inherent complexity and the fact that both are often studied at different scales^2^. While climate is studied at global and regional scales, water quality is more often studied locally.

Both urban and rural pressures on water quality are subject to the most rigorous analysis based on monitoring and policy review regimes with cost-benefits scrutinized over defined time-scales^3,4^. Agricultural diffuse pollution of water resources caused by losses of agro-chemicals and manure (including nutrients and pesticides) from land to water, is a particularly challenging and on-going issue. This is due in part to the complex rainfall-runoff pathways between soil surface sources in the agricultural landscape and receiving water bodies^5,6^. The issue widens to actor-stakeholder expectations of investment in mitigation actions at the most local source scale (*viz*. the farm), which may ultimately constrain agricultural production nationally and internationally^7^. The expectation on improving water quality at larger scales may, however, be hindered by the i) changes in agricultural practices and intensity (*e.g*. increased livestock density)^8,9^, and effective levels of actions implemented, ii) temporal chemical patterns (concentration and its variability) in stream water, in relation to monitoring periods and catchment response times^10^, iii) knowledge of catchment-related processes driving water quality^11^ and associated hydrological time lags (months to years) and biogeochemical (years to decades) time lags^12^, degradation and recovery, including human activities^13^, and iv) changes in weather^14^. The four parameters are clearly linked^2^ and have consequences within review frameworks. Despite being driven by climatic factors, these last two factors are often not explicitly taken into account when analysing changes and effects on water quality.

Here, as part of this challenge, we examine the possible links between inter-annual weather cycles and water quality^2^ considering phosphorus (P) as dissolved reactive phosphorus (DRP) or total reactive phosphorus (TRP) and nitrogen (N) as nitrate in runoff from agricultural land, with regard to their importance for drinking water quality and risk of eutrophication^15^. We specifically investigate the interactions between the local catchment scale and larger, oceanic scale climate and weather patterns.

We acknowledge that hydrometeorological processes drive P and N diffuse pollution and are linked to mobilisation processes which, at the landscape scale, are related to land use and soil nutrient management and status^16^. Such complexity ordinarily prevents the decoupling of agricultural effects from climate effects^14^. We limit our study to agricultural catchments in Western Europe for two reasons. Firstly, European Union (EU) and European Economic Area (EEA) countries are subject to diffuse pollution management and reviews under the EU Water Framework Directive (WFD – OJEU, 2000), associated daughter directives and national legislations. This provides a common policy framework of options of measures and targets. Secondly, we integrate results from three western European seaboard countries that are subject to larger scale oceanic climate and weather patterns and have high temporal resolution water quality monitoring records of diffuse pollution data in agricultural catchments defined as long term research observatories (Table 1). Such high temporal resolution monitoring is required to capture the full dynamics of nutrient loss to water over the year, without being skewed to representative sampling events and periods (baseflow/event flow, summer/winter etc.)^17^ and to cover the full range of agri-environmental conditions.

**Table 1:** Catchments characteristics. Annual precipitation, river discharge and runoff coefficient were averaged for hydrological years (1^st^ October 2009 – 30^th^ September 2015).

Catchment	Longitude	Latitude	Size	Soil	Precipitation	Discharge	Runoff	Sampling frequency	
			[km^2^]	drainage	[mm/yr]	[mm/yr]	coeff.	RP	Nitrate-N
**Ireland:**									
Corduff	−6° 51′ 1″	54° 1′ 48″	3.3	Poor	1085	625	0.57	Sub-hourly	Sub-hourly
Dunleer	−6° 24′ 49″	53° 50′ 6″	9.5	Mod.	935	445	0.48	Sub-hourly	Sub-hourly
Cregduff	−9° 10′ 29″	53° 36′ 48″	31.2*	Well	1460	185*	—	Sub-hourly	Sub-hourly
Ballycanew	−6° 18′ 51″	52° 37′ 7″	11.9	Poor	1080	520	0.48	Sub-hourly	Sub-hourly
Castledockerell	−6° 34′ 56″	52° 34′ 5″	11.2	Well	1020	550	0.54	Sub-hourly	Sub-hourly
Timoleague	−8° 46′ 11″	51° 37′ 47″	7.6	Well	1120	620	0.55	Sub-hourly	Sub-hourly
**Norway:**									
Mørdre	11° 24′ 4″	60° 6′ 34″	6.8	Poor**	737	375	0.51	14 days***	14 days***
Skuterud	10° 49′ 52″	59° 41′ 6″	4.5	Poor**	1004	643	0.64	14 days***	14 days***
Skas-Heigre	5° 47′ 21″	59° 39′ 0″	28.3	Well	1270	656	0.52	14 days***	14 days***
Time	5° 40′ 46″	58° 43′ 46″	1.1	Well	1369	857	0.63	14 days***	14 days***
Vasshaglona	8° 27′ 33'	58° 20′ 7″	0.9	Well	1470	1074	0.73	14 days***	14 days***
**France:**									
Kervidy-Naizin	−2° 49′ 52″	48° 0′ 20″	5.0	Well	910	351	0.39	Daily + storms	daily
Moulinet	−1° 11′ 50″	48° 36′ 42″	5.0	Well	878	343	0.39	Daily + storms	n/a

*Estimated spring contribution zone, **extensively tile drained, ***volume proportional composite samples.

The overall aim of the study was to investigate the influences and relationships between weather processes amplified by the North Atlantic Oscillation (NAO) during a sharp upward trend (2010–2016) and the patterns of diffuse P and N losses to waters across highly monitored catchments in Ireland, Norway and Northwestern France. The objectives were to 1) identify a catchment typology based on characteristics prone to NAO amplifications of diffuse pollution, 2) identify the relationship between NAO*i* and nutrient concentration trends in streams draining agricultural land, and 3) highlight policy issues for recovery and source management, taking into account these climatic and weather effects.

While anthropogenic warming has influenced weather in Western Europe, for example as observed by an increased number of days with extreme winter rainfall in southern England^18^, weather patterns and trends in this region are largely influenced by large-scale climate systems over the North Atlantic^19^. The NAO is one of the most prominent systems, caused by differentials in the Icelandic low and Azores high pressure systems, and its intensity can be expressed with an index (NAO*i*)^20^. In Northwestern Europe, a positive phase in the NAO*i* is often associated with elevated air temperatures in summer and more pronounced rainfall with more frequent large rain events in winter than normal^20,21^. This variation between summer and winter can influence soil drying and wetting periods, and consequently, influence soil chemical lability at the same time as increasing the magnitude and frequency of runoff events. Multi-decadal variability in ocean circulation can be twice as large as the background anthropogenic warming^22^ and the effect of weather cycles change may be regionally amplified^23^. Additionally the response of summer temperatures to anthropogenic warming in Europe is likely influenced by the same processes that cause variations in the position of the summer storm track^24^, *i.e*. large-scale climate systems over the North Atlantic.

## Methods

### Weather analysis

Regional weather amplifications were investigated by comparing daily rainfall and air temperature from the 1960s (when the intensity of the NAO was low) with recent data (2009–2015), when the NAO intensified and the time when Europe has developed datasets for the evaluation of current measures for mitigating N and P loss to water. The influence of a rise in NAO*i* on weather was analysed by correlating annual average temperatures, annual total amounts of rainfall and the annual number of large rain events (exceeding 10 and 25 mm).

### Hydrochemical analysis

Nutrient loads from catchments are highly influenced by and positively correlated to rainfall-to-runoff processes^25^. Nutrient concentrations in rivers are more subtly influenced by an integration of weather influences, including rainfall-to-runoff processes, rain (or wetness) periodicity^26^ and temperature controlled microbial soil nutrient cycling^27^. We therefore compared five to seven hydrological years of annually averaged reactive P and nitrate-N concentrations (hydrological years, 1^st^ Oct – 30^th^ Sep) in stream water (aggregated from sub-hourly monitoring and bi-weekly samples, n = 26 to 35,040) to the average NAO*i* for the same period and time step, provided by the NOAA Climate Prediction Centre (www.noaa.gov), for six catchments in Ireland, five in Norway and two in France (Table 1). The number of analysed years reflects those with available data in each catchment during the period when a sharp rise in NAO*i* occurred (2010–2016). A simple linear regression analysis of reactive P (RP) and nitrate-N concentrations to NAO*i* and levels of significance was conducted in SigmaPlot 11.0 (Table 2). A Cook’s distance analysis was conducted to identify potential outliers and influencers. If outliers could be explained by an influential occurrence that was not related to the changed weather conditions, they were omitted from the regression analysis. The NAO*i* data were in many cases filtered by antecedent simple moving averages (SMA), equally weighting 3 to 5 antecedent years with the current year. This was to avoid temporal delay, such as the yearly differences in time lags of nutrient mobilisation and transfer to stream water in monitored rivers, as well as other influences such as the Gulfstream, which may hide the correlation between RP and nitrate N concentrations with NAO*i*.

**Table 2:** Linear regression analysis of Reactive P and Nitrate-N concentrations in rivers to NAO*i*.

Catchment	Reactive P = b_o_ + b_1_*NAO*i*								Nitrate-N = b_o_ + b_1_*NAO*i*							
	SMA	n	b_0_	b_1_	R^2^	t	p	u	SMA	n	b_0_	b_1_	R^2^	t	p	u
	[yr]	[yr]							[yr]	[yr]						
**Ireland:**																
Corduff	4	6	0.028	−0.007	0.88	74.38	0.005	0.001	4	6*	1.163	0.146	0.03	10.37	0.737	0.223
Dunleer	4	7*	0.106	−0.024	0.18	16.68	0.341	0.013	4	7	4.991	−0.016	0.00	14.43	0.990	0.696
Cregduff	5	5	0.032	0.053	0.79	6.65	0.043	0.001	5	7*	1.356	−0.012	0.00	4.98	0.991	0.464
Ballycanew	0	6**	0.068	0.011	0.95	124.57	<0.001	0.001	0	7	2.533	0.395	0.28	20.13	0.222	0.321
Castledockerell	0	7*	0.028	−0.001	0.01	16.48	0.799	0.004	0	7*	7.017	0.965	0.87	95.68	0.002	0.187
Timoleague	4	7	0.074	0.066	0.82	19.04	0.005	0.008	0	7	5.838	0.965	0.57	32.20	0.049	0.423
**Norway:**																
Mørdre	3	6**	0.060	0.058	0.99	73.54	<0.001	0.002	3	7	3.045	0.071	0.00	9.80	0.945	0.699
Skuterud	4	7	0.050	−0.053	0.64	9.96	0.031	0.010	4	7	3.780	0.613	0.23	26.41	0.279	0.288
Skas-Heigre	4	6*	0.032	−0.050	0.83	9.18	0.012	0.006	0	6*	3.119	−0.636	0.45	18.56	0.146	0.385
Time	0	7*	0.080	0.004	0.02	15.71	0.742	0.013	4	7	5.000	1.915	0.76	36.43	0.011	0.276
Vasshaglona	4	7	0.070	0.026	0.20	10.90	0.308	0.013	0	7*	4.275	0.497	0.17	19.76	0.354	0.552
**France:**																
Kervidy-Naizin	5	7*	0.055	0.124	0.74	6.32	0.014	0.015	3	7	13.193	−6.892	0.91	42.84	<0.001	0.693
Moulinet	0	6	0.024	0.016	0.49	6.00	0.122	0.009	n/a	n/a	n/a	n/a	n/a	n/a	n/a	n/a

*Analysis includes an outlier year as defined by Cook’s D test > 1. **Analysis excludes an outlier year as defined by Cook’s D test > 1.

Antecedent simple moving averages (SMA) used for best correlation, number of analysed years (N), intercept (b_0_), slope (b_1_), t value (t), correlation coefficient (R^2^), probability (p) and uncertainty of the regression (u).

### Hydrochemistry and weather data

In the Irish catchments, stream water level was recorded on a 10-min basis using OTT Orpheus Mini vented-pressure instruments installed in stilling wells in all catchments outlets. River discharge was calculated *via* rating curves developed (in WISKI-SKED software) on Corbett flat-v non-standard weirs at the catchment outlets using the velocity-area method with OTT Acoustic Doppler Current meters. Unfiltered TRP concentrations were monitored concurrently in all catchments outlets using a Hach-Lange Sigmatax-Phosphax suite of instruments. It was assumed that TRP was approximately equivalent to DRP since the flow-weighted mean DRP was previously reported to account for 98–99% of the flow weighted mean TRP in these catchments^28^. The bankside P analysers^29,30^ continuously measure TRP (3–4 measurements per hour) by colorimetry using the molybdate-antimony method. The measuring range for TRP is 0.010 mg l^−1^ to 5.000 mg l^−1^ and the detection limit is 0.010 mg l^−1^. Total oxidised N (TON) was monitored in the outlet on a sub-hourly basis using Hach-Lange Nitratax SC-Plus UV instruments (0.1 – 50 mg l^−1^). It was assumed that TON was equivalent to nitrate-N based on low nitrite-N concentrations^31^. Weather data was provided by Met Éireann, the Irish Meteorological Service (www.met.ie/).

In the Norwegian catchments hourly average, maximum and minimum discharge were calculated based on water levels, recorded at the catchment outlets every 10 s using a pressure transducer connected to a Campbell data logger, and site-specific rating curves. Composite water samples were collected automatically in the outlets on a volume proportional basis in all catchments^32^. The composite water sample in the container represented the average concentration during the sampling period. The composite water samples were collected for analysis every 14 days, but more frequently during periods with high runoff conditions. Heating-lamps or -cables together with more frequent maintenance was used in winter to prevent ice formation in the monitoring station, thereby guaranteeing year round reliable discharge measurements. Filtered samples (0.45 µm) were used to determine DRP spectrophotometrically by the ammonium molybdate method^33^, with ascorbic acid as a reducing agent and detection limit 0.002 mg l^−1^. Nitrate-N was determined according to standard methods (EPA-600/4-79-020) with a detection limit of 0.002 mg l^−1^. Results below the detection limit were estimated to be 50% of detection limit. Weather data was provided by the Norwegian Meteorological Institute (www.eklima.no).

In the French Kervidy-Naizin catchment, the stream water level was recorded at the outlets of the catchments every minute using a Thalimèdes OTT float-operating sensor while in the Moulinet catchment a Starflow Ultrasonic Doppler Instrument was used to record stream water level and velocity every 10 minutes. River discharge was calculated *via* rating curve developed for Kervidy-Naizin and for Moulinet by multiplying the current velocity by the wetted area. To obtain consistent 10 min frequencies, the 1 min measurements in Kervidy-Naizin were subsampled every 10 min. Two monitoring strategies complemented each other for P concentrations acquisition in order to sample both base and storm flows: regular sampling every 1 to 6 days (manual in Kervidy-Naizin; automatic in Moulinet) at approximately the same time (17:00 local time), and high-frequency sampling during storm events with autosamplers (ISCO 6712 Full-Size Portable Sampler). The autosamplers were placed in the shade at the outlet and collected samples when the stream water level reached a threshold, at a frequency of one sample every 30 min for 12 h. Samples were filtered (0.45 µm cellulose acetate filter) directly on-site and sent to the laboratory within 1 week for determination of DRP colorimetrically by reaction with ammonium molybdate with a precision of ±0.004 mg l^−1^. In Kervidy Naizin another aliquot of each stream water sample was filtered directly on site to 0.22 µm for determination of nitrate concentration by ionic chromatography (DIONEX DX 100) with a precision of ±2.5%. Weather data was provided by Meteo France (www.meteofrance.com/accueil).

Despite differences in monitoring strategies, all three were designed to provide highly accurate annual P and N concentrations based on sample collection across all flow ranges.

## Results and Discussion

### Times of change

**Figure 1:**
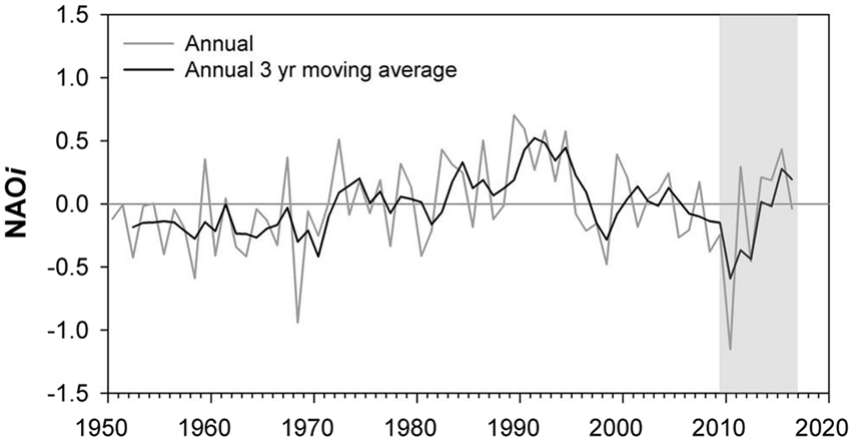
Time series of the North Atlantic Oscillation index (*NAOi*) provided by the NOAA Climate Prediction Centre (www.noaa.gov). The lines illustrate the annual average and annual antecedent simple three-year moving average *NAOi*, and the shaded area represents the period for high resolution monitoring of phosphorus and nitrogen concentrations in rivers interpreted in this study (2009–2016).

During the last approximate decade, there has been a distinct upward trend in the NAO*i* (Fig. 1) and this has occurred with other records of regional meteorological change in the study areas. This may have offset both hydrological and chemical baselines in Western European Rivers. In Ireland, particularly influenced by the Atlantic Ocean^22^, the 1981–2010 mean annual precipitation over the country was 5% higher and the mean annual air temperature 0.5 degree higher compared with the 1961–1990 average^34^. There were, however, regional variations in precipitation and evidence of an increase in the number of days with heavy rain (10 mm or more)^34^. In Norway, annual average precipitation and air temperatures have increased since 1900^35^. While precipitation increased more in spring and less in summer, temperature increased more in spring and less in winter^35^. In France, the recent period of positive NAO*i* concurred with an increase in winter rainfall, decrease in spring and autumn rainfall and increase in annual temperature^36^. These national trends were also observed in the western part of France with a more pronounced seasonal distribution^36^.

**Figure 2:**
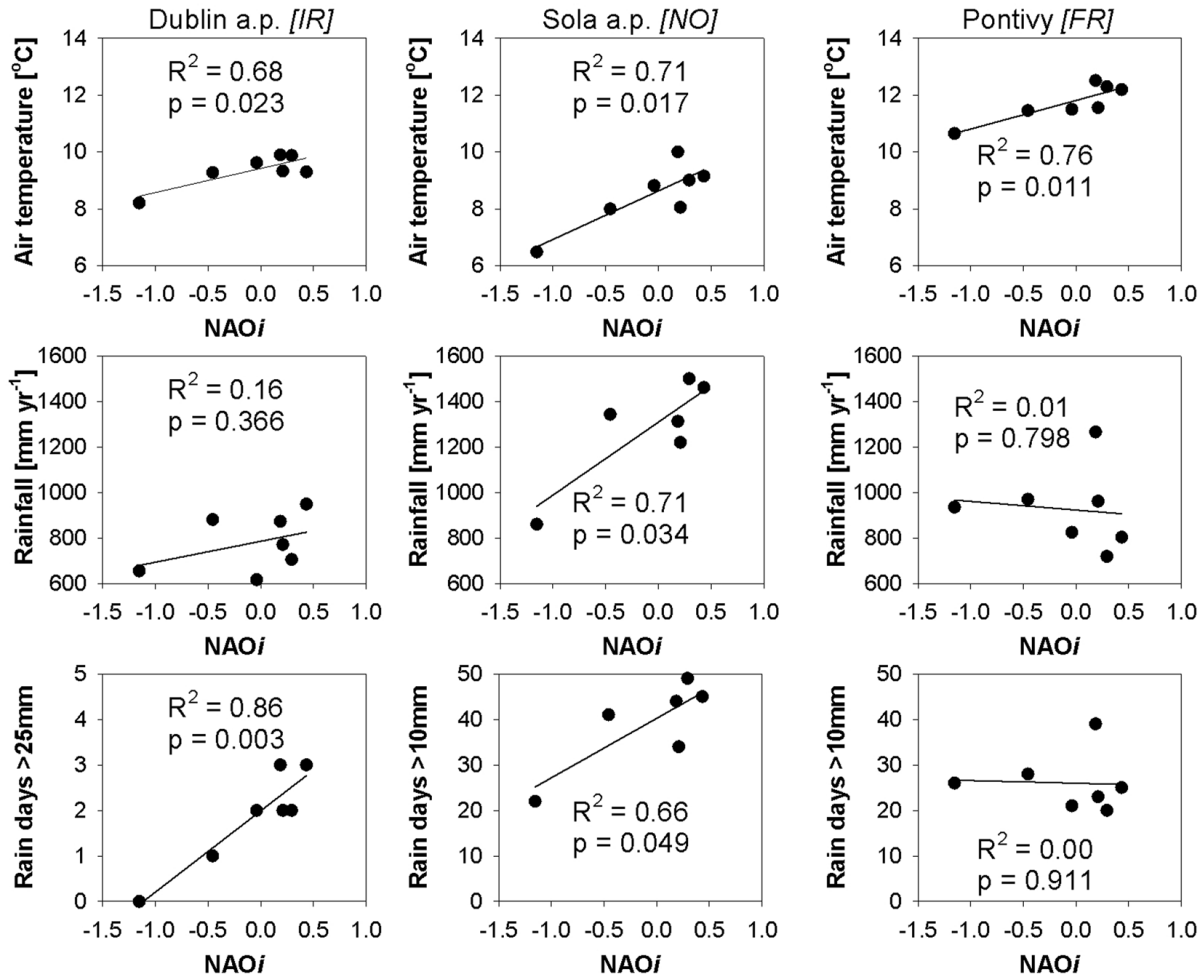
Examples of the impact of a sharp rise in NAOi to annual mean air temperature (top), annual total rainfall (middle) and annual number of rain days exceeding 10 mm or 25 mm (low) correlated to NAO*i* for three meteorological stations, Dublin airport (Met Éireann, Ireland), Sola airport (Norwegian Meteorological Institute, Norway) and Pontivy (Meteo France, France) for the period 2010–2016.

During the monitoring period, coinciding with the sharp rise of NAO*i* (2010–2016), there was a significant and positive correlation of annual mean air temperature to the NAO*i* (Fig. 2) in three national weather stations, one in each country (close to the catchments analysed in this study), with the largest change in the Norwegian station. The annual total rainfall was only significantly positively correlated to the NAO*i* in the Norwegian station while the number of large rain events correlated positively to NAO*i* in both the Irish and Norwegian stations (Fig. 2). In the Irish station the number of rain days exceeding 25 mm correlated to NAO*i* and in the Norwegian station the number of rain days exceeding 10 mm correlated to NAO*i*, while in the French station there was no correlation of number of rain days exceeding either 10 or 25 mm.

Warmer summers and more frequent large rain events in winter affect soil P and N lability and subsequent mobilisation and transfer processes. Average total P flux and concentrations normally increase with higher rainfall volume and intensity^37^ and variations in weather have previously explained observed changes in both stream N fluxes and concentrations^14^. Such changes can impact negatively on aquatic ecology by causing enhanced algal blooms^38^. Further, the position of the Gulf Stream, responding to variations in El Niño Southern Oscillation (ENSO) and NAO*i*^39^, was found to influence lake N concentrations in Ireland, with implications for both quantity and quality of biota^40^.

### The NAO and nutrient delivery

Warmer summers and increase in heavy winter rains, responding to the sharp rise in NAO*i*, are divided into two main theoretical categories to explain potential trends of P and N concentrations in rivers.

1. Warmer summers and more rain or more frequent winter storms, as potentially expressed by a positive and larger NAO*i*, can increase P concentration in streams of headwater catchments if: (i) P is mobilised (erosion^41^ and soil biochemistry^42^) and (ii) surface flow pathways are dominating (conditions controlling flow)^5^, or (iii) below ground transfer dominate but are not attenuated (subsurface flow controls)^43^, or (iv) soil P sources are directly connected to the stream (landscape and management). If P is retained or P sources are not hydrologically connected, larger NAO*i* will likely decrease P concentration due to dilution or delay the process. That would also be a likely scenario if the main source of P is from a persistent point source^8^. Nitrate is highly mobile and its concentration may increase by an increased below-ground transfer of nitrate-rich groundwater volumes towards the river, and by shorter residence times induced by more runoff (more shallow groundwater tables, higher hydraulic gradients and quicker pathways activated) and therefore less denitrification along the whole transfer continuum, or decrease by: (i) dilution of surface flow paths (if there is high N storage in the groundwater or if there are long residence times) and/or (ii) denitrification due to more saturation.
2. An enhanced summer mineralisation can increase P and N lability at the start of the wet period^44^. In this case P and N concentration will increase with higher surface runoff and leaching with the first rains after summer^45,46^ (but not if there is high storage potential in the groundwater or long residence times causing attenuation)^47^.

**Figure 3:**
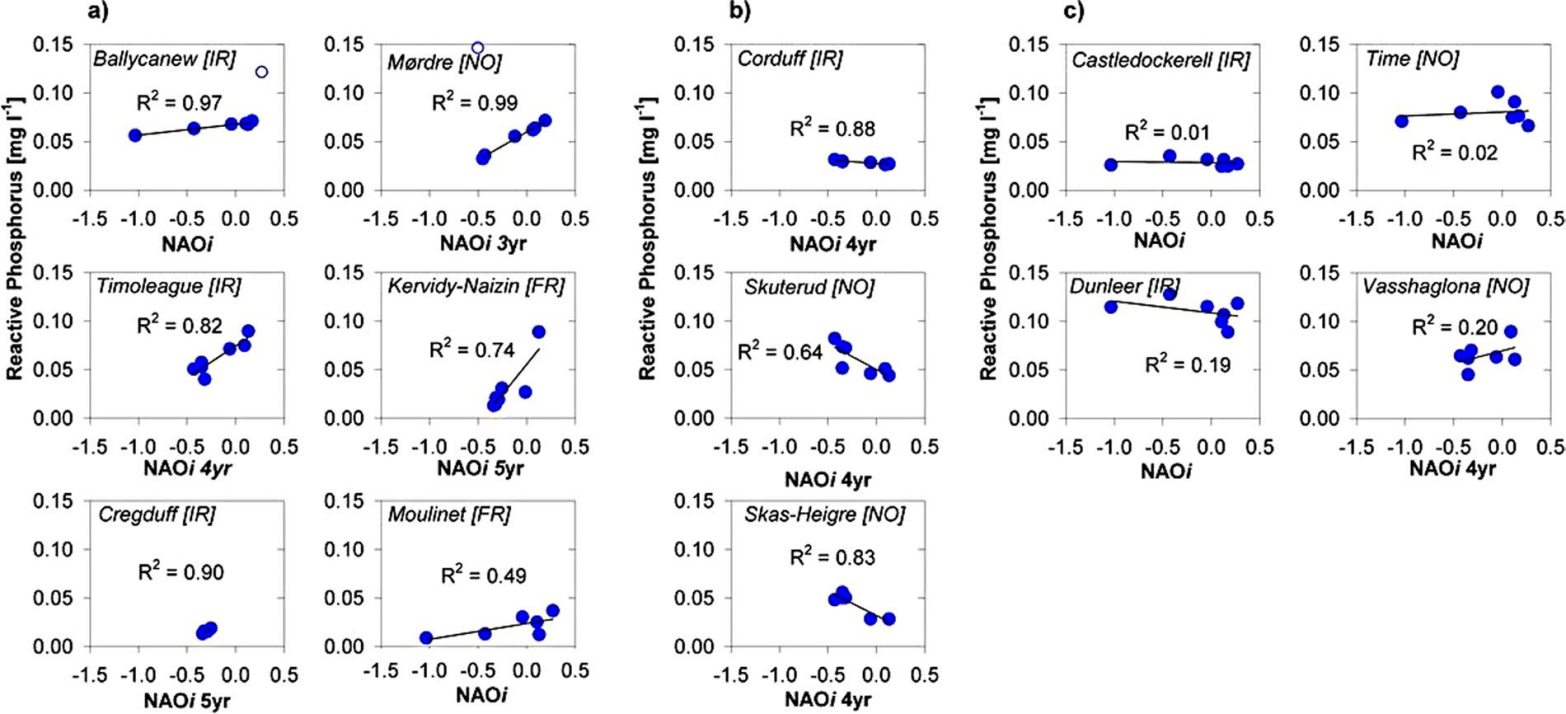
Linear correlation of Reactive P concentrations in rivers to NAO*i*. Annual averages (hydrological years, October - September) of reactive phosphorus concentrations correlated to NAO*i*: (**a**) positive correlations, (**b**) negative correlations and (**c**) no correlations in Irish, Norwegian and French catchments with different flow controls, farm management, weather response to NAO change, P retention and storage capacity. NAO*i* are expressed as antecedent zero to five-year simple moving averages depending on the specific catchment response time.

**Figure 4:**
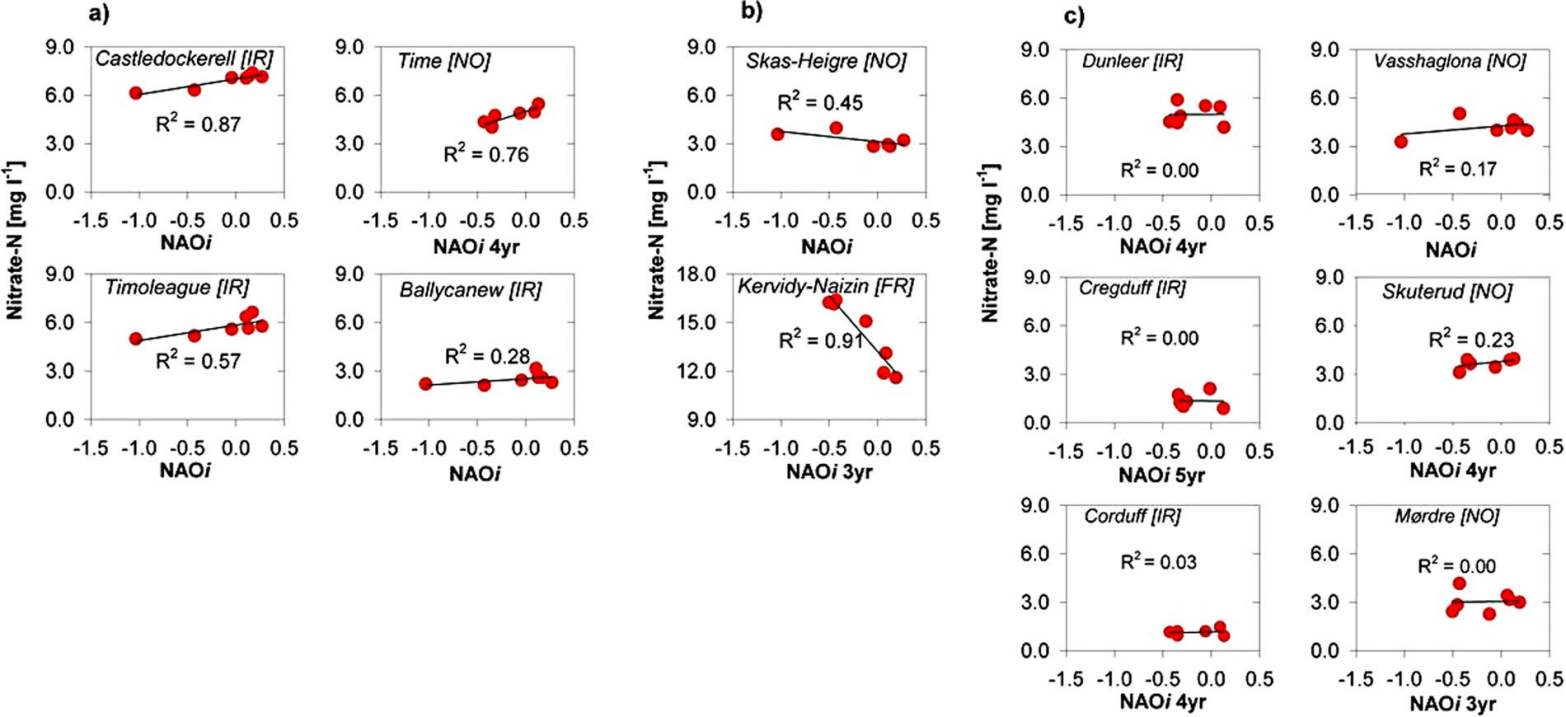
Linear correlation of nitrate-N concentrations in rivers to NAO*i*. Annual averages (hydrological years, October - September) of nitrate-N concentrations correlated to NAO*i*: (**a**) positive correlations, (**b**) negative correlations and (**c**) no correlations in Irish, Norwegian and French catchments with different flow controls, farm management, weather response to NAO change, N retention and storage capacity. NAO*i* are expressed as antecedent zero to five-year simple moving average depending on the specific catchment response time (there is no N concentration data for the Moulinet catchment).

Integrating this theoretical framework with high temporal resolution nutrient and discharge data composited into annual averages (hydrological years that fully incorporate storm event data), both N and P (as nitrate-N and Reactive Orthophosphate) concentrations were in many cases (P: 10/13; N: 8/12) correlated to NAO*i* (Fig. 3a–c, Fig. 4a–c and Table 2). The degree of correlation was viewed in terms of the two categories described above (mobilisation, transfer pathway, storage, attenuation, hydrological connectivity and dilution). The influence of changes in fertilizer application to the fields of the catchments was seen to be of minor importance for P and N concentrations during the analysed period and further dampened by the release of legacy P and N^48^. In some catchments, P and N concentrations were positively correlated to the NAO*i* (P: R^2^ = 0.49–0.99 (n = 6) and N: R^2^ = 0.28–0.87 (n = 6)) and in other catchments there were negative correlations (P: R^2^ = 0.64–0.88 (n = 4) and N: R^2^ = 0.45–0.91 (n = 2)), poor or no correlation (P: n = 4 and N: n = 6). Since the catchments response may present potential time-lags of losses to stream water, NAO*i* was in those catchments expressed as an antecedent three to five-year moving averages to achieve a stronger correlation between annual NAO*i* and N and P concentration. Reasons for this time-lag could involve the dominating nutrient mobilisation processes, transfer pathways and catchment size. With this method, we highlight catchment ‘memory’ effects. This was particularly the case for the Norwegian catchments as explained by the strong influence of the Gulf stream^49^ at which latitude correlates to the NAO*i* with a two-year delay^21^. The results first present RP then nitrate-N, and for each, considering positive and then negative correlation.

### Positive correlation, RP

Two catchments, Ballycanew and Mørdre, both have poorly drained soils and P was likely mobilised through erosion and lost to surface water via surface runoff. Reactive P may be desorbed from the eroded soil particles in the stream bed, and in these catchments the annual average RP concentrations were positively and strongly correlated to NAO*i* (Fig. 3a: Ballycanew, R^2^ = 0.97 (excluding an outlying year with highly elevated RP concentration in baseflow conditions, possibly due to point-based sources) and Mørdre (R^2^ = 0.99) (excluding an outlying year with intensive rain after sowing and fertilizer application, resulting in the highest registered mean RP concentration during the monitoring period)). Both of these catchments have flashy hydrology (high ratio of storm flow to base flow magnitudes), steep slope gradients (particularly Mørdre) and highly erodible soils^50,51^ resulting in a large proportion of RP desorbed from mobilised soil particles resulting from rainfall to runoff partitioning influences^5,50^ and a positive correlation of runoff to NAO*i*. The P concentrations in the rivers of these catchments were therefore highly and positively related to the NAO influence.

The four catchments Timoleague, Creggduff, Kervidy-Naizin and Moulinet are dominated by well-drained soils and mostly groundwater-fed flow. The RP concentrations in these catchments appeared sensitive to weather shifts (Fig. 3a and Table 2) due to leaching and transfer *via* groundwater^43^. It was likely that soil chemistry and enhanced mineralisation influenced the mobilisation of P in these catchments, and therefore also the correlation of RP concentration and NAO*i*. The Timoleague, Kervidy-Naizin and Moulinet catchments have soils with high soil P status, and are iron and carbon rich (with subsequent P sorption competition), favouring P mobilisation into soluble form and leaching to shallow groundwater^42,43,52^. Alternatively, RP is directly exported from highly saturated areas, consequently responding by an increased RP concentration with more rain as hydrological gradients are elevated (R^2^ = 0.82, 0.74 and 0.49 respectively). However, since the increase in NAO*i* had influenced the mean air temperature but not the annual rainfall nor the frequency of high rainfall days in the French catchments (Fig. 2) a strong positive correlation of RP to NAO*i* was not expected there. There were only weak positive correlations of runoff to NAO*i* in the two Irish catchments (data not shown). In the Cregduff catchment, a karst spring contribution zone of pure limestone, P was largely retained in the aluminium rich soil and calcium rich weathered bedrock^53^. However, while a large proportion of P was retained in the karst, an excess of P was transferred to the conduits *via* slow small fissure and matrix flow where it was settled, remobilised and transferred further by larger event flows^53^. In this catchment P loss was to some degree defined by rainfall to runoff partitioning influences with a positive and strong correlation of P concentration to the antecedent five-year moving average NAO*i* (R^2^ = 0.79). However, the mobilisation and transfer time from source to delivery point may be long and more reflected by an antecedent average NAO*i* rather than annual fluxes, and the P concentrations in the rivers of these four catchments are more reflective of previously mobilised, attenuated and subsequently remobilised P – i.e. a ‘memory’ of the NAO influence.

### Negative correlation, RP

Despite a positive correlation of runoff to NAO*i* (data not shown) due to an increased rainfall (Fig. 2), there was a significant negative correlation of RP concentration and NAO*i* in the Corduff, Skuterud and Skas-Heigre catchments (R^2^ = 0.88, 0.64 and 0.83, respectively), all on poor to moderately drained soils (Fig. 3b and Table 2). The reasons for this relationship were not entirely clear and were likely to be catchment-specific. For example, in the Corduff catchment the negative correlations may be due to combinations of iron and calcium rich soils and high soil P retention in conjunction with hydro-chemical disconnection caused by natural landscape features^54^ that strengthened dilution. In Skuterud, the livestock production ceased in 2010, which may influence point source RP concentrations. Skas-Heigre are dominated by high livestock density and high soil P status, but were influenced by an official P-mitigation project from 2010. Additionally, the Skuterud and Skas-Heigre, are both equipped with sedimentation ponds to mitigate sediment and particulate P transfer and which may also, in part, act as a mode to disconnect RP transfers by slowing down the flow, allowing for retention and cause dilution. However, the hydrological conditions that increase RP transfer during extreme positive phases of NAO*i* may also act to overcome catchment dis-connectivity and such catchments will require further research with focus on specific periods. An alternative explanation could be that these two catchments have small but persistent point sources likely to be diluted by more rain, more frequent large rain events (Fig. 2) and therefore more runoff.

### No correlation, RP

The Castledockerell, Dunleer, Time and Vasshaglona catchments, all on well-drained soils, had no correlation of RP concentration to NAO*i* (R^2^ = 0.01, 0.19, 0.02 and 0.20 respectively, Fig. 3c). In both Castledockerell and Time this can be explained by high soil P retention caused by soils rich in Al with less P solubility^43,55^.

### Positive correlation, nitrate-N

An increased number of days where soils have high soil temperatures and optimal soil moisture may enhance stored (short and long-term) organic N mineralisation and nitrification^45,46^, and if this is followed by more frequent large rain events, nitrate is easily leached to groundwater and loss to the stream water is likely to occur. In such cases, if the stream is groundwater fed and the pathways are not attenuated, stream nitrate concentration would respond positively to an intensified NAO. An increased hydrological gradient would further reduce the residence time for denitrification.

The three catchments, Castledockerell, Timoleague and Time, are all dominated by well-drained soils and with mostly groundwater-fed rivers. Under such conditions excessive nitrate could be leached to groundwater and transferred to the rivers. The annual average nitrate-N concentrations were positively correlated to NAO*i* in these catchments (Fig. 4a: R^2^ = 0.87, 0.57 and 0.76 respectively). The strongest correlation was found in the Castledockerell catchment, which largely lacks denitrifying conditions and has relatively short time lags due to a thick (up to *ca*. 20 m), highly permeable layer of weathered slate bedrock^56^ and a positive correlation of rainfall and runoff to NAO*i*. The Timoleague catchment, with its permeable sandstone geology and denitrifying conditions in the near stream zones^56,57^ had a lower correlation of nitrate-N concentration to NAO*i*, even if the source pressures were stronger than in Castledockerell. The Time catchment possibly has a large proportion of shallow groundwater together with a network of tile drains transferring nitrate to the river that was largely influenced by the NAO*i*.

The nitrate-N concentration in Ballycanew catchment, with a flashy hydrology, had a weak positive correlation to NAO*i* (R^2^ = 0.28). This catchment has a transfer *via* shallow perched groundwater to the rivers. Moreover, nitrate-N is transferred to the river via a line of springs, where the upland well drained soils meet the low land poorly drained soils. Another important transfer pathway in that catchment is an extensive network of tile drains and ditches^58^ that could transfer nitrate-N relatively quickly with little attenuation, thus responding positively to changes in NAO*i*.

### Negative correlation, nitrate-N

In two of the catchments, Skas-Heigre and Kervidy-Naizin, the nitrate-N concentration responded negatively and correlated well to the increased NAO*i* (Fig. 4b: R^2^ = 0.45 and R^2^ 0.91 respectively). In Skas-Heigre, with a positive correlation of rainfall and runoff to NAO*i*, one explanation for the negative correlation is the potential mitigating effect of the sedimentation pond, which slows down the stream flow allowing for denitrification and dilution during rainfall. Furthermore, a high groundwater table in this catchment may contribute to high denitrification rates. However, this will need further testing. The Kervidy-Naizin catchment has a large N storage capacity in the groundwater which may mask the effect of leaching and during subsequent winter rain events there will be higher water flux but lower nitrate-N concentrations^57^. This catchment has been found to lack a relationship between annual N surplus and stream nitrate-N concentration due to higher temperatures and high nitrate-N fluxes from groundwater to stream, and long residence times^59^.

### No correlation, nitrate-N

The Dunleer, Cregduff, Corduff, Vasshaglona, Skuterud and Mørdre catchments had no correlation of nitrate-N concentration to NAO*i* (R^2^ from 0.00 to 0.23, Fig. 3c). In the Dunleer catchment perched shallow groundwater is the highest source for N transfer to the stream, and this is only hydrologically connected, via open ditches that are active in winter, when the groundwater table is high^60^. It was noted that seasonal N concentration amplitudes were greatest in Dunleer during wet autumns following dry summers (*i.e*. increased lability – not shown). However, the overall effect appears to be dilution during periods of increased rainfall frequency in this catchment and so the ditches acted as limited and seasonal ‘on-off switches’, hence weakening the correlation to NAO*i*. In the karst Cregduff catchment nitrate-N was likely leached and transferred via slow micro fissures to the conduits, where up to 90% of the P transfer occurred during spring flow events^53^, and with a large potential for denitrification and weakening of the influence of NAO. The Corduff and Vasshaglona catchments have shallow groundwater tables with a potential for denitrification in the near stream zones. The Skuterud and Mørdre catchments are both intensively tile drained and runoff is transferred quickly on the soil surface and via macropores to tile drains with little contact to the soil matrix and hence nitrate-N concentrations may be diluted. Furthermore, Skuterud catchment has a potential for denitrification in the sediment pond and this may also weaken the correlation to NAO*i*. This has not been shown.

### Systems science integration

Such relationships, only found by the analysis of data covering the full hydrochemical discharge range, have implications for the interpretations of data in catchments showing, for example, decreases in nutrient source pressures or implementations of other catchment mitigation measures^61^. An increase in NAO*i*, increasing the average air temperatures and causing more rain or more days with large rain events, may override these positive benefits in some years or indicate greater benefits in other years – and this is likely to be catchment-specific. A consideration of dominating mobilisation and transfer processes, storage and hydrological connectivity should, therefore, be included as moderating factors in policy reviews of diffuse pollution management.

Using the oceanic scale NAO*i* and local scale water quality data is an example of a ‘climate-chemical indicator’ in the framework of integrating systems-science^1^. However, planning for diffuse pollution mitigation, when some years will indicate smaller or greater benefits against a baseline of measures, may still leave water quality and agricultural actors and stakeholders with a conundrum. That is: how to integrate these chemical-climate systems further with socio-political indicators of environmental improvement against a back drop of expectations and targets. Furthermore, another integration is the need to sustain agricultural production for economic and food security. Over-engineering mitigations by increasing catchment resilience in vulnerable catchments to account for positive phases of NAO*i* (or other weather amplifications) or accepting phases of failed or exceeded targets will leave certain elements of the overall integrated system wanting. Developing a deeper understanding of the economic consequences of, for example, over-engineering or optimizing all elements of the systems related to catchment-specific diffuse pollution may be a solution that is more readily understood and implemented^62,63^. At the very least, developing climate-chemical indicators into catchment vulnerability indicators will provide a new and important component to water quality management objectives in agricultural catchments.

## Conclusions

Oceanic-scale climate patterns, characterized by the intensity of the North Atlantic Oscillation (NAO*i*), was found to influence nutrient loss to water to a varying degree in agricultural river catchments in the northwest of Europe (Ireland, Norway and North Western France). A sharp upward trend in NAO*i*, which has amplified annual weather patterns in recent time, may override or underride benefits of local management, in a specific way in some catchments and years. A new and important component to diffuse pollution and water quality management objectives is therefore to develop integrated climate-chemical indicators into catchment response indicators. While simple mean residence time is often mentioned, it is also needed to consider the hydro-biogeochemical sensitivity of the catchments to climate variations.
